# Attentional bias to threat is modulated by stimulus content: an fNIRS study

**DOI:** 10.3389/fnhum.2023.1308457

**Published:** 2024-01-11

**Authors:** Hejun Liu, Qihan Zhang, Jon D. Elhai, Christian Montag, Haibo Yang

**Affiliations:** ^1^Academy of Psychology and Behavior, Faculty of Psychology, Tianjin Normal University, Tianjin, China; ^2^Department of Psychology, University of Toledo, Toledo, OH, United States; ^3^Department of Psychiatry, University of Toledo, Toledo, OH, United States; ^4^Department of Molecular Psychology, Institute of Psychology and Education, Ulm University, Ulm, Germany

**Keywords:** attentional bias, eye gaze, functional near-infrared spectroscopy (fNIRS), threat type, attentional bias modification (ABM)

## Abstract

People are evolutionarily predisposed to associate threat relevant stimuli with fear or aversiveness and show an attentional bias toward threat. Attentional bias modification (ABM) has been shown to reduce threat biases, while quantitative reviews assessing the effectiveness of bias modification yielded inconsistent results. The current study examined the relationship between the training effect of attentional bias to threat and the type of threatening stimuli. Twenty-two participants performed a modified dot-probe task while undergoing functional near-infrared spectroscopy (fNIRS) imaging. Results indicated that there was a strong pattern of attentional avoidance among individuals in an animal but not human threat condition. Furthermore, findings from fNIRS confirmed that the influence from type of threatening stimulus would be modulated by cortical activation patterns, especially in the ventrolateral prefrontal cortices (vlPFC) and angular gyrus. Overall, these results suggest that stimulus-specific may play a major role in personalization of specific psychological interventions.

## Introduction

Being able to rapidly detect and react to threat-relevant information was of critical importance for the survival of humans and likely has a deep evolutionary origin (Mogg and Bradley, 1998; Panksepp, 1998; Montag et al., 2013; van Rooijen et al., 2017). Preparedness theory has postulated that humans are evolutionarily predisposed to associate threat-relevant stimuli with fear or aversiveness (Seligman, 1971; Ohman and Mineka, 2001). This perceptual prioritization of threat was an adaptive mechanism to prepare humans for acting upon the imminent danger to mobilize defensive resources promptly and avoid potential harm (Carlson et al., 2016). See also analysis of the fear system in different theories ranging from Gray (McNaughton and Corr, 2004; Reuter et al., 2015) to Panksepp et al. (2011), LeDoux (2014), and Montag and Davis (2018).

The claim for attentional bias for evolutionarily relevant threat has received an impressive amount of empirical support under a variety of experimental paradigms, conditions and clinical populations (Bar-Haim et al., 2007; Notebaert et al., 2011). Several studies examining visual search processes have reported that pictures of snakes and spiders elicit faster response times than other objects (e.g., flowers and mushrooms) in both humans (adults, infants and children) (Ohman et al., 2001; Lobue and DeLoache, 2008; Soares et al., 2009; LoBue, 2010; Soares and Esteves, 2013) and non-human primates (Shibasaki and Kawai, 2009). Similarly, human performance on the dot probe task has also found that other threats apart from predatory and venomous animals, including images of threatening faces and bodily harm, could also capture attention (Koster et al., 2006; Carlson et al., 2009). As can be seen, a similar pattern of attentional bias was found for both animal and human threat-relevant stimuli. These findings support the animate monitoring hypothesis, which proposed that humans are predisposed to attend preferentially to live objects in the environment (New et al., 2007). At the neural level, evidence from functional MRI studies have identified several brain regions that also respond greater to threat stimuli than for threat-irrelevant stimuli (Lacreuse et al., 2013). One of the neuroanatomical models was the stimulus-driven system associated with the amygdala, implicated in the emotional processing and directing attention to threatening information in the environment (Frewen et al., 2008; Carlson et al., 2016). Beyond this it is well known that the defensive distance between predator and prey play an important role to activate the fear system in the latter group (Mobbs et al., 2007). More importantly, attentional bias to negative-stimuli may arise from perturbation of a top-down attentional control system mediated by the ventrolateral and dorsolateral portions of the prefrontal cortices (vlPFC; dlPPFC) (Price et al., 2014; White et al., 2016; Edvinsson et al., 2017; Sylvester et al., 2017).

This selective attention to threat has been reliably observed across a range of anxious and other clinical populations, and characterizes healthy people with a vigilance particularly to highly threatening stimuli (Wilson and MacLeod, 2003; Koster et al., 2004a,b; Waters et al., 2004; Bishop, 2008; Kappenman et al., 2015; Pintzinger et al., 2016). Mounting studies suggest that the aberrant deployment of attention to negative emotional information may be a causal factor in the proximal illness process (Beck, 1976; Browning et al., 2010a; Mogg et al., 2017). Besides, these biases can be altered using cognitive training tasks (also known as attentional bias modification: ABM) (MacLeod and Mathews, 2012), confirmed in both clinical and non-clinical populations (Browning et al., 2010b). There is growing evidence that the use of ABM threat-avoidance training (e.g., the dot-probe task as the most common paradigm) in various clinical populations ameliorates symptoms [e.g., see reviews by Heeren et al. (2015), Li et al. (2016), Mogg et al. (2017), Kuckertz et al. (2019), Hang et al. (2021)], and the training effect in non-clinical participants also led to a significant decrease in attention to negative stimuli (MacLeod et al., 2002; Eldar et al., 2008; See et al., 2009; Browning et al., 2010b). Some quantitative reviews have examined the efficacy of ABM threat avoidance training on attentional bias, revealing that ABM produced a large effect in early analyses (Hakamata et al., 2010), while more recent reviews have suggested that ABM produced a small effect or was even ineffective (Cristea et al., 2015a,b; MacLeod and Clarke, 2015).

Such inconsistency in the training effect was not an isolated finding. One possible explanation for the instability of training effects relates to the different types of stimuli used by researchers (Ghosn et al., 2019). Studies have been conducted to examine the differential effects of aversive (e.g., threatening words or images) or appetitive (e.g., alcohol, smoking, food) stimuli on ABM (Beard et al., 2012). However, to our knowledge, no direct studies have examined whether the effectiveness of ABM training on attentional biases universally to all types of threats or is differentially influenced by threats from animals (e.g., spiders and snakes) and human (e.g., mutilation and blood) in the same dot-probe paradigm. Recent research has reported that faces (or bodies, etc.) that contain social information have a greater spontaneous attentional capture effect (van Rooijen et al., 2017). In addition, some studies have indicated that the reduction of change in attentional bias achieved with ABM tasks may be a consequence of improved attention control mediated by the prefrontal cortex (Mogg et al., 2017; Mogg and Bradley, 2018), such as via increased activation of the lateral prefrontal cortex (lPFC) (Browning et al., 2010b; Clarke et al., 2014; Nelson et al., 2015), while eye gaze may induce a faster attentional orienting and an amplified performance facilitation for targets by generating an internal representation of the spatial location indicated by the cue (Bindemann et al., 2007; Zhao and Zhang, 2009; Kuhn and Tipples, 2011; Hornung et al., 2019). Accordingly, for this study, a modified version of the dot-probe task with an eye gaze cue was designed to maximize the change in attention away from threat and enhanced attentional control using the blood oxygenation level-dependent (BOLD) functional near-infrared spectroscopy (fNIRS) signal. Therefore, we predicted that individuals would show different patterns of attentional bias for both animal and human threat stimuli, and may even recruit inconsistent neural circuits during the ABM training.

## Materials and methods

### Participants

A total of 24 college students participated in the study for monetary payment. All participants were right-handed and did not have brain injury or a history of mental disorder. This study was approved by the Ethics Committee of Tianjin Normal University. Each participant was informed about the general nature of tasks and stimuli prior to signing an informed consent form.

### Apparatus

An fNIRS system (LABNIRS/16, Shimadzu Corporation, Kyoto, Japan) with a 3-wavelength (780 nm, 805 nm and 830 nm) near infrared semiconductor laser system (1M level under the IED-60825-1 standard) was used at a sampling rate of 10 Hz in this study. Based on the modified Beer-Lambert law (MBLL), changes in the concentrations of oxy-hemoglobin (HbO), deoxy-hemoglobin (HbR) and total hemoglobin (HbT) were obtained by measuring the changes in near-infrared light absorption after its transmission through the tissue. Hoshi et al. (2001) and Hoshi (2007) showed that HbO is a sensitive indicator of the change in regional cerebral blood flow during task simulation and has a higher signal-to-noise ratio than HbR and HbT. Thus, our study focused on the HbO concentration only as an analysis indicator.

### Stimuli

Eighty pictures from the Chinese Affective Picture System (Bai et al., 2005) were selected: 48 neutral images that included scenery, 32 unpleasant images that depicted threat (animal: 16; human: 16). All pictures were cropped and resized to 433 × 325 pixels by a photo editing software (Adobe Photoshop). A total of 25 additional college students revaluated these pictures using a 9-point Likert-type scale on valence and arousal. The results showed a difference in the valence rating of neutral (*M* = 5.40, SD = 0.42) and threatening (*M* = 2.66, SD = 0.74) images. In terms of arousal, threatening scenes (*M* = 6.78, SD = 0.77) were rated as high-arousing images compared to neutral scenes (*M* = 4.24, SD = 0.38). Besides, we examined the differences in valence and arousal across types of threat stimuli. The results of paired-sample *T*-test revealed both threat-stimulus categories did not differ in either valence [*t*(15) = −0.32, *p* = 0.75, *Cohen’s d* = −0.08] and arousal [*t*(15) = 0.41, *p* = 0.68, *Cohen’s d* = 0.09].

**FIGURE 1:**
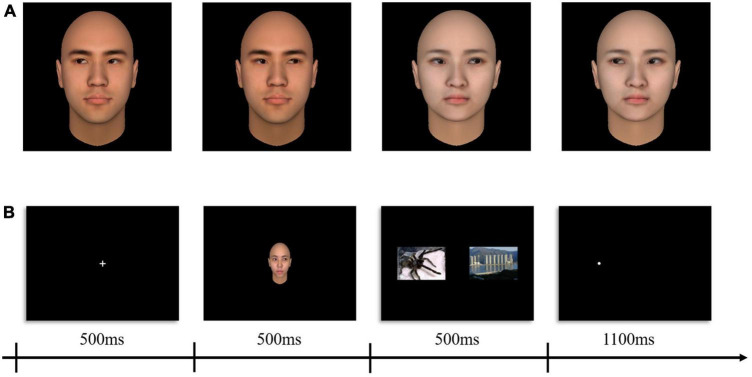
Sample social attention stimuli and schematics of the procedure. **(A)** Examples of adapting stimuli (left gaze = –100%, right gaze = + 100%). **(B)** Example trials sequence of experimental paradigm.

We created 4 neutral faces (2 man; 2 women) as social attention (gaze cue) stimuli using FaceGen Modeller.^1^ Two gaze directions (maximally left and maximally right) for each face were generated in FaceGen (see Figure 1A).

### Procedures

This study adopted a within-subject design with two within-subject factors: probe location (congruent vs. incongruent) and stimuli type (human threats vs. animal threats). During fNIRS, prior to and after the experimental procedure, participants were, respectively, given 60 s to rest. The formal experiment (modified dot-probe task) was presented to participants using E-prime software (2.0, Psychology Software Tools, Inc., Sharpsburg, PA) and displayed on a 13-inch monitor (Resolution: 1,024 × 768; Refresh rate 60 Hz). We employed an event-related design in which the complete presentation process of one trial was shown in Figure 1B. Trials comprised a centered fixation crosshair for 500 ms, social attention cue (the gaze direction always toward the location of the neutral images) for 500 ms, immediately afterward images in pair (neutral - threatening) appeared for 500 ms, a white dot probe (0.5 × 0.5 cm) appeared either on the left or the right side of the display and remained visible until 1,100 ms had elapsed. Participants were instructed to identify the position of the dot probe by pressing the “f” or “j” keys on the keyboard quickly and accurately. Each stimulus type was presented several times leading to a total of 96 trials. A total of 48 null trials (displaying only a plus sign and requiring no response) were first included in a randomized order to establish a baseline condition. Stimuli were presented in a pseudo-randomized fashion. A trial was labeled congruent when the dot probe appeared at the location of the emotional images and was labeled incongruent when the dot probe appeared at the location of the neutral images (see Figure 1B). Variable jitter times (4,000–9,000 ms) as intertrial interval was set to reduce temporal adjustment of the subjects to the task (Emberson et al., 2015).

### Probe arrangement

**FIGURE 2:**
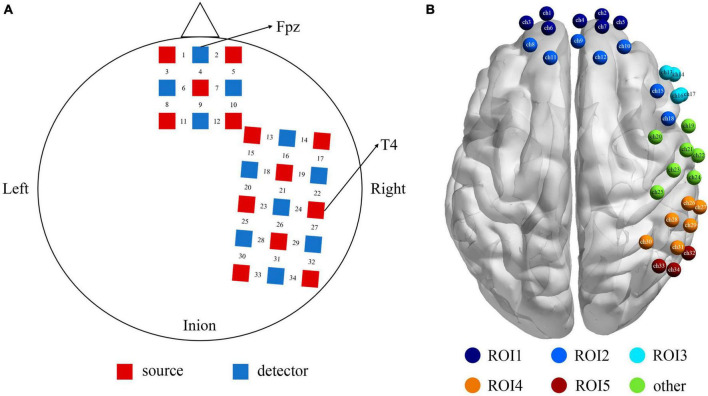
**(A)** The positions of the optode probes, the red squares represent the sources, and the blue squares represent the detectors, and between a source and a detector is the channel. **(B)** The functional localization into 5 ROIs based on proximity of channels and anatomy. ROI1 (the purple) = Frontopolar cortex (ch 1, 2, 3, 4, 5, 6, 7), ROI2 (the dark blue) = dlPFC (ch 8, 9, 10, 11, 12, 15, 18), ROI3 (the sky blue) = vlPFC (ch 13, 14, 16, 17), ROI4 (the orange) = SupraMarginal gyrus (ch 26, 27, 28, 29, 30, 31), ROI5 (the red) = angular gyrus (ch 32, 33, 34), and the rest channels in green (ch 19–25) were not the area of interest for this study.

One “3 × 3” and one “3 × 5” multi-channel probe board was used, including 13 emitters and 11 detectors, with a 3 cm distance between probes, forming a total of 34 channels. For each participant, the Fpz and T4 set as the reference point were placed on the bottom central and the rightmost central probe, respectively, according to the international 10–20 EEG system (see Figure 2A). Furthermore, the 3D Locator (FASTRAK; Polhemus, Colchester, VT, USA) was used to obtain the 3D coordinate points and channel anatomical locations based on head landmarks (Cz, Nz, AL, and AR). The probabilistic registration method was used for registration in the NIRS-SPM system of each channel position and Montreal Neurological Institute (MNI) spatial coordinates to obtain the corresponding Brodmann areas (see Supplementary Table 1 for further details). Our choice to cover the right hemisphere was based on prior studies that there was an overall pattern with the relationship between the right hemisphere and negative expression (Maxwell et al., 2005).

Our montage was designed to optimize coverage of brain structures in the frontal, temporal, and parietal lobes. Figure 2B shows the functional localization clusters. Given the current data on neural correlates of attentional bias process in both healthy and clinical populations (White et al., 2016; Sylvester et al., 2017), we focused on five ROIs, that is, the Frontopolar cortex (FPC), dlPFC (dorsolateral prefrontal cortex), vlPFC (ventrolateral prefrontal cortex), SupraMarginal gyrus (SMG), and angular gyrus.

### Data analysis

Cerebral cortex blood oxygenation data were processed with the NIRS-Statistical Parametric Mapping (NIRS-SPM) toolbox (version; v.4.1) operated based on MATLAB (Ye et al., 2009). Noises (head movement, heart rate, etc.) and drifts of the extracted fNIRS signal were eliminated by the Hemodynamic Response Functions (HRF) and wavelet-minimum description length method (Jang et al., 2009). The degree of the reaction induced by the experimental tasks in response to the reference wave (beta value) on each channel was evaluated by the general linear model (GLM) to obtain the fitting coefficient β, and the temporal autocorrelation of this process was adjusted using the pre-coloring method (Worsley and Friston, 1995).

Two participants were excluded from the sample for the following reasons: large motion artifacts and failure to remain awake during the process of data collection, and therefore, the final analysis dataset comprised 22 participants (14 women, mean age = 21.2 ± 1.7 years). Paired-samples *t*-tests of the obtained beta values according to the experimental design were used to investigate if there was activation under different task conditions. The significant level was set at *p* < 0.05. False discovery rate (FDR) correction was used to minimize false positive results (Noble, 2009). Limited by the number of sample size, we adopted permutation testing (5,000 iterations) to verify the statistical significance (Collingridge, 2013).

## Results

### Behavioral data

**FIGURE 3:**
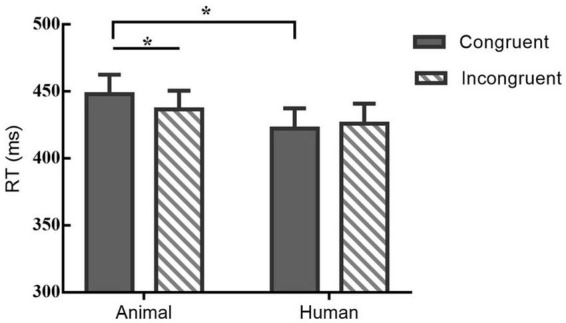
Mean RT (bars show standard errors) under different probe and stimuli type; *indicates significant differences between.

Response times (RTs) of very short duration (<100 ms) or timeouts (>1,000 ms) were discarded and we analyzed only trials with correct responses (Joormann and Gotlib, 2007). In order to explore the pattern of attentional bias under the effect of eyes gaze and examine the role of stimulus type, two-factor repeated measures ANOVAs were conducted on the behavioral data. For the RT, there was a significant main effect of stimuli-type, *F*(1,21) = 5.131, *p* < 0.05, *η^2^* = 0.20. Although the main effect of probe location on RT was not significant [*F*(1,21) = 1.415, *p* > 0.05, *η^2^* = 0.06], there was a trend of higher mean congruent type than the incongruent type. Besides, there was a significant interaction between probe-location and stimuli-type on RT, *F*(1,21) = 5.798, *p* < 0.05, *η^2^* = 0.22. A *post hoc* pairwise comparison using the Bonferroni correction showed a shorter RT of the incongruent than congruent condition under the animal stimuli [*t*(21) = −2.19, *p* < 0.05, *Cohen’s d* = −0.32], however, for the human stimuli participants’ RT formed no significant difference between the two locations of probe [*t*(21) = 1.02, *p* = 0.32, *Cohen’s d* = 0.11]. Table 1 and Figure 3 illustrates the results of RT.

**TABLE 1:** Mean (with standard deviations) of RT (ms) under different probe location and stimuli type.

Type	Animal stimuli	Human stimuli
Congruent	447.95 ± 66.72	422.44 ± 68.80
Incongruent	436.72 ± 63.67	426.12 ± 67.83

### Near-infrared spectroscopy data

**FIGURE 4:**
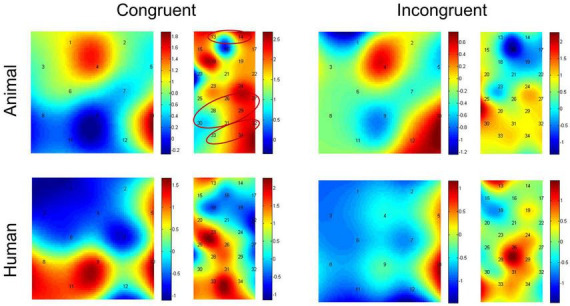
Heap maps of *t* values comparing activations between baseline and probe location in both the animal and human stimulus types. The *t* values at the optode probe location are determined through nearest-neighbor interpolation of the surrounding channels, and the data for the plot is further generated by cubic interpolation. Solid circles indicate the ROIs that showed significantly increased activations.

Paired-samples *t*-tests between baseline and probe location for each of the stimulus types were used to investigate if there was activation during the dot-probe task, and the t value for each channel in different conditions can be found in Figure 4. The average activation of the channels representing each ROI was further analyzed. For the animal stimuli type, there was significantly higher brain activation in the congruent condition in the vlPFC, *t*(21) = 2.295, *p* = 0.032 (marginal significance after FDR corrected), the SupraMarginal gyrus (SMG), *t*(21) = 2.529, *p* = 0.019 (FDR corrected), and the angular gyrus, *t*(21) = 2.829, *p* = 0.010 (FDR corrected) compared with the baseline. No ROI areas were significantly activated when in the incongruent condition (*ps* > 0.05). However, for the human stimuli type, no ROI areas were significantly activated compared with baseline in any location of probe (*ps* > 0.05). We have also re-analyzed these data with permutation test, and found results remain the same (Congruent condition of animal stimuli type: *p* = 0.016 for vlPFC, *p* = 0.011 for SMG, *p* = 0.007 for angular gyrus; *p* > 0.05 for all other conditions).

**FIGURE 5:**
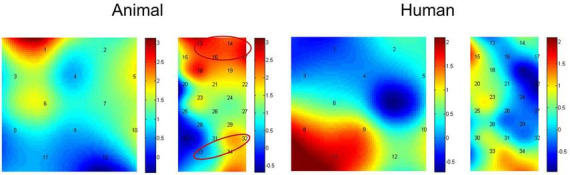
Heap maps of *t* values comparing activation differences between the congruent condition and incongruent condition in both the animal and human stimulus types. The *t* values at the optode probe location are determined through nearest-neighbor interpolation of the surrounding channels, and the data for the plot is further generated by cubic interpolation. Solid circles indicate the ROIs that showed significantly increased activations.

In addition, we then compared activation differences between the congruent condition and incongruent condition using paired-samples *t*-tests, and the t value for each channel in both the animal and human stimulus types can be found in Figure 5. The average activation of the channels representing each ROI was further analyzed. Results showed the animal stimulus type evoked significantly higher activation in the congruent than incongruent condition in the vlPFC, *t*(21) = 3.608, *p* = 0.002 (FDR corrected), and the angular gyrus, *t*(21) = 2.180, *p* = 0.041 (marginal significance after FDR corrected). There was no significant difference found among probe location under human stimulus type (*ps* > 0.05). We have also re-analyzed these data with permutation test, and found results remain the same (Animal stimuli type: *p* = 0.001 for vlPFC, *p* = 0.018 for angular gyrus; *p* > 0.05 for all other conditions).

We also analyzed the HbR data. The preprocess is the same way as the HbO data, and the paired-samples *t*-tests also showed the significant activation difference between probe location in the vlPFC and the angular gyrus (*ps* < 0.05, FDR corrected) under animal stimulus type. However, there was no significant difference found among baseline and probe location under animal stimulus type (*ps* > 0.05, FDR corrected).

### Neural-behavior relation

To connect the significant findings in vlPFC and angular gyrus to behavior, we then tested the partial correlations between the differences of probe location [Bias Index (BI) = incongruent trials - congruent trials] (Hornung et al., 2019) of brain activation and behavioral performance after controlling for age and gender. For the vlPFC and angular gyrus the correlations were not significant. For the SupraMarginal gyrus (partial *r* = −0.500, *p* = 0.025), a negative association emerged under the human stimulus condition, but not for the animal stimulus condition. And the same trend was found in the frontopolar cortex for both animal (partial *r* = −0.511, *p* = 0.021) or human (partial *r* = −0.533, *p* = 0.016) stimuli conditions, respectively.

## Discussion

The current study aimed to evaluate the neural mechanism implicated in the training effect of attentional bias to threatening stimuli, and the possible modulation by stimulus type (animal threats vs. human threats) in a healthy adult sample using a modified dot-probe task with a within-subject design. The present study also provides preliminary evidence for stimulus-specific modulation that would allow the personalization of specific psychological interventions, resulting in greater effectiveness of bias modification.

Behavioral results provide evidence for cueing effects of gaze that may contribute to the modification of attentional bias toward threatening stimuli. Besides, there may be possible existence of two separate mechanisms for the modification of attentional bias across threat stimulus type. Specifically, participants responded significant slower to congruent than incongruent conditions, and this pattern of attentional avoidance only occurred within the condition of animal threats, which was not found in human threats. These findings in the human threats condition are consistent with the previous literature that humans and rhesus monkeys share mechanisms of social attention that allow the rapid detection of threatening conspecifics in the environment (Lacreuse et al., 2013). Yet, an important finding of this study is that attentional biases to conspecific threatening stimuli were stronger than non-conspecific threatening stimuli in humans. The differences can be due to multiple mechanisms. Specifically, the detection of different threats is influenced by evolutionary mechanisms (Carlson et al., 2016) and experience (LoBue, 2010). However, subjects’ greater sensitivity to human threats relative to animal threats may be due to the regulatory mechanisms of amygdala-prefrontal recruitment even under increased attentional control (Lacreuse et al., 2013; Carlson et al., 2016).

Brain imaging results further supported our hypothesis that stimulus type would modulate cortical activation patterns. When the animal threats were introduced, we found stronger involvement of the vlPFC and the angular gyrus during congruent compared to incongruent trials, while no significant neural difference was found between congruent and incongruent trials under the human threats condition. A likely explanation for this finding may be found in the theory of biased competition models of attention, which argues that captured attention may be achieved by using the lateral prefrontal cortex (LPFC) mechanisms to “prime” or strengthen the representations of stimuli that occur at a given location or other factors (Bishop et al., 2004; Bishop, 2008). Among them, the vlPFC region is crucially involved in attentional control processing (Price et al., 2014; White et al., 2016; Edvinsson et al., 2017; Sylvester et al., 2017) and thus greater vlPFC activation during animal stimulus presentation may reflect augmented top-down control to support processing of the attended stimuli influenced by the gaze-cueing effect (Bishop, 2008; Frewen et al., 2008; Browning et al., 2010b; Fani et al., 2012; Li et al., 2012). That is, improvement of attentional control can result in the avoidance of animal threats. Besides, the angular gyrus (BA 39), a subregion of the temporoparietal junction (TPJ), also played an important role in shifting of attention to allocate attentional resources to more efficiently process information from a stimulus (Friesen et al., 2004, 2005; Krumbholz et al., 2009). However, we did not find the same pattern of activation in the human stimulus condition, which may indicate consistency and combined with our behavioral results, demonstrates that there was a significant effect of stimulus-specific to attentional bias training.

In addition, negative correlations were revealed between the neural and behavioral bias scores in SupraMarginal gyrus (SMG) in the human stimulus condition and also in Frontopolar (FPC) in the arbitrary stimulus condition, suggesting that an increased behavioral bias index reflects a greater tendency of individuals’ attention to be drawn by human threats stimuli with social information (van Rooijen et al., 2017), with a concomitant reduction in the potential neural correlate (Lacreuse et al., 2013; Hornung et al., 2019). The region of SMG (BA 40) was considered to mainly constitute key nodes of the ventral attention network, which play a pivotal role in detecting unattended or unexpected stimuli and triggering shifts of attentional, leading to the allocation of more attentional resources to emotional stimuli (human threats) (Corbetta and Shulman, 2002; Hutchinson et al., 2009; Vossel et al., 2014). Previous studies showed that significantly decreased connectivity in anterior insula and SMG after ABM training (Li et al., 2016). Taking this a step further, the finding in the present study might therefore suggest that the attentional bias of human threats was also moderated by SMG. Besides, SMG has been shown to be important for empathy (Vollstädt-Klein et al., 2019). Human empathy for conspecific threatening stimuli compared to the non-conspecific may add evidence to our study of differences in response across stimulus type. The Frontopolar (BA 10), which is responsible for attentional control and was correlated in both stimulus conditions (Godier et al., 2016; Hilland et al., 2020), could be further illustrated that human threats stimuli were influenced to some extent by gaze cues, but this cueing effect was smaller than the emotional modulation of threat stimuli.

Several limitations of the current study need to be mentioned. First, although fNIRS is a feasible alternative to fMRI and with better spatial resolution and less susceptibility to head movement compared to EEG, it is also limited in its ability to measure hemodynamic changes in the deep brain. Further investigation of the relationship with the amygdala-PFC system may be useful for understanding the mechanism under stimulus-specific conditions during attentional bias training. Second, we used animal and human stimuli within the same task, and the two types of threat stimuli may mutually affect each other either through a priming effect or by increasing arousal (Lacreuse et al., 2013). Therefore, caution in interpretation is required when presenting both animal and human threat stimuli. Third, it has been hypothesized that over age, the pattern of attentional bias toward threats changes (van Rooijen et al., 2017). Future work with larger sample sizes across various age groups should continue to investigate possible age-related bias in reliability. Besides, Zhai et al. (2020) suggest that placement errors may be unavoidable due to the lack record of subject’s head size, and the tapered contrast vector method is better than uniform contrast vector method in ROI analysis, so further studies should take head circumferences (e.g., as covariates) and the new methodology in ROI analysis (tapered contrast vector) into account to confirm the present findings.

To conclude, the current study provides evidence that animal, but not human, threat stimuli elicit a strong pattern of attentional avoidance in healthy Chinese participants, mainly due to the cueing effect of gaze. And the neural basis further confirmed that the influence of the type of threat stimulus was modulated by cortical activation patterns, especially in the vlPFC and angular gyrus associated with the control and shift of attention. The results extend our understanding of the mechanisms of stimulus-specific conditions as a major contributor to the inconsistency of the training effect of attentional bias in individuals and may enable personalization of specific psychological interventions.

## Data availability statement

The raw data supporting the conclusions of this article will be made available by the authors, without undue reservation.

## Ethics statement

The studies involving humans were approved by the Tianjin Normal University, China. The studies were conducted in accordance with the local legislation and institutional requirements. The participants provided their written informed consent to participate in this study.

## Author contributions

HL: Conceptualization, Data curation, Formal analysis, Methodology, Visualization, Writing—original draft. QZ: Writing—review and editing. JE: Writing—review and editing. CM: Writing—review and editing. HY: Conceptualization, Writing—review and editing.

## Footnotes

1
https://facegen.com/
