# Supplementary material for: Attentional bias to threat is modulated by stimulus content: an fNIRS study
Source: Front Hum Neurosci. 2024 Jan 11;17:1308457. doi: 10.3389/fnhum.2023.1308457 (PMC10808614; doi:10.3389/fnhum.2023.1308457)
Supplement: Supplementary file 1 [file Table_1.DOC]

Supplementary Material

Table S1. Channels and corresponding brain regions.

| Channel | Anatomical Label | Overlap(%) |
| --- | --- | --- |
| CH01 | 10-Frontopolar area | 0.96 |
| CH02 | 10-Frontopolar area | 0.89 |
| CH03 | 10-Frontopolar area | 1 |
| CH04 | 10-Frontopolar area | 1 |
| CH05 | 10-Frontopolar area | 0.98 |
| CH06 | 10-Frontopolar area | 0.89 |
| CH07 | 10-Frontopolar area | 0.95 |
| CH08 | 9/46-Dorsolateral prefrontal cortex | 0.93 |
| CH09 | 9-Dorsolateral prefrontal cortex | 0.75 |
| CH10 | 9/46-Dorsolateral prefrontal cortex | 0.95 |
| CH11 | 9-Dorsolateral prefrontal cortex | 0.98 |
| CH12 | 9-Dorsolateral prefrontal cortex | 1 |
| CH13 | 45-ventrolateral prefrontal cortex | 0.91 |
| CH14 | 45-ventrolateral prefrontal cortex | 0.74 |
| CH15 | 9/46-Dorsolateral prefrontal cortex | 0.57 |
| CH16 | 45-ventrolateral prefrontal cortex | 0.85 |
| CH17 | 45-ventrolateral prefrontal cortex | 0.85 |
| CH18 | 9-Dorsolateral prefrontal cortex | 0.57 |
| CH19 | 6-Pre-Motor and Supplementary Motor Cortex | 0.57 |
| CH20 | 6-Pre-Motor and Supplementary Motor Cortex | 0.76 |
| CH21 | 6-Pre-Motor and Supplementary Motor Cortex | 0.56 |
| CH22 | 43-Subcentral area | 0.88 |
| CH23 | 4-Primary Motor Cortex | 0.51 |
| CH24 | 4-Primary Motor Cortex | 0.52 |
| CH25 | 4-Primary Motor Cortex | 0.55 |
| CH26 | 40-SupraMarginal gyrus | 0.71 |
| CH27 | 40-SupraMarginal gyrus | 0.99 |
| CH28 | 40-SupraMarginal gyrus | 0.54 |
| CH29 | 40-SupraMarginal gyrus | 1 |
| CH30 | 40-SupraMarginal gyrus | 0.42 |
| CH31 | 40-SupraMarginal gyrus | 0.82 |
| CH32 | 39-Angular gyrus | 0.73 |
| CH33 | 39-Angular gyrus | 0.57 |
| CH34 | 39-Angular gyrus | 1 |
